# The enhanced photocatalytic performance of CPAA doping with different concentrations of Titanium oxide nanocomposite against MB dyes under simulated sunlight irradiations

**DOI:** 10.1038/s41598-024-61983-7

**Published:** 2024-06-04

**Authors:** Marwa M. Sayed, Abdelaziz M. Aboraia, Yara A. Kasem, Nancy N. Elewa, Yasser A. M. Ismail, Kamal I. Aly

**Affiliations:** 1 Chemistry Department, Faculty of Science, New Valley University, El-Kharja, 72511 Egypt; 2 Physics Department, Faculty of Science, Al-Azhar University, Assiut, 71542 Egypt; 3 Energy Storage Research Laboratory (ESRL), Physics Department, Faculty of Science, Al-Azhar University, Assiut, 71542 Egypt; 4 Polymer Research Laboratory, Chemistry Department, Faculty of Science, Assiut University, Assiut, 71516 Egypt; 5 Physics Department, Faculty of Science, Ain Shams University, Cairo, 11566 Egypt; 6 Department of Physics, Faculty of Science, Islamic University of Madinah, Madinah, Saudi Arabia

**Keywords:** CPAA, Photocatalysis, Titanium oxide, Methylene blue, Energy gap, Environmental sciences, Materials science

## Abstract

The pure conjugated polyarylene azomethine (CPAA) and its nanocomposites (CPAA-TiO_2_) with different concentrations of TiO_2_ nanoparticles were successfully prepared by in-situ technique and analyzed by different advanced techniques. XRD has confirmed the structural properties and crystallinity of (CPAA) and nanocomposites. The SEM clearly shows that the (CPAA) is uniform and homogeneous, with tightly connected aggregate layers in shape. However, the amount of TiO_2_ in the nanocomposites greatly affects their morphology, revealing structural differences and indicating a reaction between (CPAA) and TiO_2_, especially at a higher concentration of 5% TiO_2_. A new composite of (CPAA) was introduced and the photocatalytic effect for MB was studied. The removal efficiency of (pure-CPAA) over MB dye under simulated sunlight was 62%. However, (CPAA-TiO_2_ 1%) destroyed 90% of MB dyes. It was discovered that the low band gap of (CPAA-TiO_2_ 1% (2.84 eV)) accelerates high electron–hole recombination, increasing photocatalytic activity.

## Introduction

Much interest has been shown in conducting polymers (CPs), as they have conjugated double bonds, making them a potentially attractive material^1^. The conductivity of CPs is affected by several factors, such as their doping level, dopant type, conjugation length, and interchain interactions, and thus can display either semiconducting or metallic behavior^2^. Their primary benefits include low cost, simple synthesis, high electrochemical and electrical activity, and high carrier mobility. Even though chemical synthesis is a straightforward process, the most crucial step is establishing an excellent structure–property relationship corresponding to desirable practical qualities. It has many possible monomer functionalities and reaction conditions, but regulating the final polymer's structure and molecular weight is challenging^3^. Conjugated polymers with nanostructured materials are promising new materials for solar and fuel cells. However, the study of their photocatalytic efficiency and effectiveness is still limited. Industrial applications still face difficulties with photocatalysis using solar energy to purify water by degrading organic contaminants at a lower cost and energy consumption^4^. Natural water contamination by organic contaminants^5^ has long been recognized as an urgent environmental problem due to its dangers to aquatic life and human health at ng/L to μg/L concentrations. Drugs^6,7^, insecticides, cleansing byproducts^8,9^, and other synthetic chemicals are a few examples of the huge variety of organic contaminants that are often not controlled despite their increasing importance^10,11^. As a result of progress in analytical recognition methods, the list of these substances is growing or generated as byproducts of water and wastewater treatment processes. Traditional methods of wastewater and water purification fail to eliminate these pollutants.

For decades, scientists have worked to improve the photocatalytic efficacy of existing materials by discovering and inventing new photocatalysts^12^. Photocatalysis performance can be increased by designing semiconductor interfaces, and this system could be formed by linking two inorganic semiconductors or compositing inorganic- polymer for water treatment^13^. Titanium dioxide (TiO_2_) nanoparticles and their composites are the most popular for environmental cleanup; however, technological obstacles restrict large-scale applications of TiO_2_ nanoparticles in water treatment^14–16^. The primary drawbacks of TiO_2_ as a photocatalyst are its wide bandgap, which necessitates high-energy UV light, its low photoresponse, and the challenges associated with recovering nanoparticles after their initial treatment^17,18^. Changing the surface area, sizes, forms, and surface characteristics of TiO_2_ can boost its photocatalytic efficiency^19^. Chemical alterations, such as adding components to the TiO_2_ structure or modifying the surface with conductive polymers, have been used to increase photoactivity^20,21^. Organic polymers with the Schiff base structure, like poly azomethine, polytriazine, polyheptazine, etc., are used as photocatalysts in water purification, improving photoelectric charge separation and the absorption of visible light^22–24^.

A thiazine substance known as methylene blue (MB) is regularly manufactured and utilized in multiple industries for various purposes, such as dyeing cotton, wool, and fabrics; coloring paper; using it as a hair colorant; and indicating redox reactions in space, among others. The dangers of MB to human health become apparent when it is ingested; it may harm the central nervous system and the eyes. In addition to the aforementioned symptoms, you may also have gastritis infections, nausea, vomiting, respiratory issues, and diarrhea^25,26^. So, it's critical to find ways to remove these dangerous contaminants from water sources. It is extremely desired to remove MB, a frequent carcinogenic pollutant, from the aqueous medium. MB dye is an active species in the Ultraviolet–Visible (UV–Vis) spectrum and has typical peaks in this spectrum. Identifying MB in any aqueous system is easy using UV–Vis spectroscopy since it is a colored dye that becomes blue when oxidized. So, It's also easy to determine that the MB is present in any medium because its blue color fades when it reacts with radiation-activated photocatalytic nanomaterials (NMs) in the medium. This makes it easy to keep track of the rate of the degradation reaction. Also, by observing the UV–Vis spectra, we can see that the characteristic absorption peaks of MB fade away as the MB degrades^27,28^. The photocatalytic conversion of MB dye into water, carbon dioxide, and other species like ammonium, nitrate, or sulfate ions is further proof of the degradation. So, using the photocatalysis technique, the poisonous MB dye is transformed into far less dangerous by-products^29^.

In this study, we successfully synthesized a new conjugated polyarylene azomethine CPAA and its nanocomposites with different ratios of TiO2 nanoparticles; in a trial to prepare a novel polymer with extended conjugation by a simple method to be used as a new photocatalyst with low energy band gap and also modification of its photocatalytic efficiency by using TiO2 nanoparticles the most popular environment cleaner to be used in MB photodegradation. several distinct analytical approaches characterize the generated monomer, polymer, and nanocomposites. Furthermore, the photocatalytic efficacy of these nanocomposites is assessed, and it is observed that the photocatalytic behaviour of the TiO2 semiconductor is improved by incorporating that conductive polymer.

## Experimental

### Materials

Terephthalaldehyde (Aldrich, Germany), potassium hydroxide (KOH), 4-aminacetophenone (Alfaser), titanium dioxide (TiO_2_, P-25 nm), tetrahydrofuran (THF, Aldrich), ethyl alcohol absolute (EtOH, abs.), Methylene blue dye (MB), and acetone are all pure chemicals that can be used directly.

### Measurements

The KBr method is utilized to conduct Fourier transform infrared spectroscopies (FT-IR) measurements using Shimadzu 2110 PC scanning spectrometers. Nuclear magnetic resonance (^1^H-NMR) and (^13^C NMR) spectra are collected using a JEOL (ECA 400) spectrometer using CDCl_3_ as a solvent. Powdered samples are used in X-ray diffraction crystallography (XRD) to examine their crystalline nature through a scan-type experiment. Detector SSD160 coupled in fast mode (2ϴ) and PSD (1D model). The TA Q-600 Thermal Analyzer is used to perform thermogravimetric analysis (TGA) at a heating rate of 10 °C/min in a nitrogen gas atmosphere. The Shimadzu mini 1240 is utilized for the collection of UV–Vis spectra. Using a coating technique, the Jeol JSM-5400 LV scanning electron microscope (SEM) and JEM 100 CXII transmission electron microscope (TEM) can identify the surface morphology of the specified polymers. To conduct X-ray photoelectron spectroscopy (XPS), an X-Ray000-400um- FG spectrometer was used. BET surface area (BELSORP-miniX) was measured using the nitrogen adsorption–desorption technique at a degassing temperature of 77.35 °C.

### Synthesis of conjugated azomethine monomer (CA)^30^

Under N_2_ gas, in a 250-mL flask with continuous stirring and a condenser, terephthaldehyde (1.5 g, 1 mol) in about 25 ml of ethanol (abs.) and 4-aminacetophenone (3.023 g, 2 mol) in 40 ml of ethanol (abs.) were dissolved together and warmed at 50 °C for 3 h. A pale yellow solid is precipitated from the reaction mixture, filtered, and repeatedly washed with hot ethanol to obtain yellow crystals in 70% yield (m.p. 215 °C). ^1^H NMR (400 MHz, CDCl_3_): δ 2.50 (s, (CH_3_) 6H_a_), 7.33–8.05 (aromatic proton ArH), 8.39 (s,(CH=N) 2H_b_). ^13^C NMR (CDCl_3_) δ 197.09, 160.6, 155.89, 138.65, 134.99, 129.77, 129.45, 120.87, 26.51. The molecular ion peak was visible in the mass spectrum at m/z = 368.2 (72.5%), consistent with its molecular formula (C_24_H_20_ N_2_O_2_).

### Synthesis of conjugated polyarylene azomethine (CPAA)

Terephthaldehyde (0.072 g) in THF (10 mL) is condensed with CA monomer (0.2 g) in 20 mL THF in a molar ratio of 1:1; KOH (0.069 g) in 5 mL THF acts as a catalyst. The catalyst was added to the monomer solution drop by drop at 65 °C under nitrogen. When the first drop of the catalyst is introduced, the reaction solution changes colour, signifying the beginning of polymer precipitate. After 24 h of continuous stirring, a yellow precipitate is generated (to ensure that all monomer molecules have had a chance to react). The polymer is then thoroughly washed with THF and distilled water to remove traces of KOH and impurities numerous times with hot ethanol. Next, the polymer is dried at 80 °C to eliminate any remaining water molecules, and a yellow polymer powder with a 75% yield is attained.

### Synthesis of CPAA/TiO_2_ nanocomposite

Synthesis of CPAA/TiO_2_ nanocomposites hybrid using KOH-catalyzed in situ condensation polymerization in the presence of TiO_2_ nanoparticles (25 nm) (with varying wt % of monomer). The common technique is that a solution of terephthaldehyde (1 mmol) in 10 ml THF is combined with a solution of CA (1 mmol) in 20 ml THF; next, TiO_2_ nanoparticles (varying wt%, 1.0%, 3.0%, and 5.0%) are added while stirring continuously. Then, 5 ml of KOH solution is added drop by drop while the mixture is held under heating conditions. After steady stirring, a solution with a brownish colouring is formed, and a yellow-coloured precipitate forms. Finally, the product was washed with THF and distilled water to eliminate residual base and hot ethanol. The product is dried overnight at 80 ºC.

### Photocatalytic activity test

Photocatalytic activities were determined by measuring MB degradation in an aqueous solution for each sample. A mixture of 1 g l^−1^ catalysts and 400 ml of a 50 mg l^−1^ MB aqueous solution was irradiated with visible light, the visible light source is 500 watts and 16 lm/W. The MB and photocatalyst suspension were magnetically agitated in the dark for 60 min to achieve adsorption–desorption equilibrium before turning the light on. Regular samples were taken from the reactor and centrifuged immediately to eliminate any solids that could have been suspended in the liquid. A UV–vis spectrometer was used to examine the clear solution. The MB concentration was determined using a calibration curve. After each round of photoreactivity testing, the suspension was centrifuged to separate the solution. The sample's photocatalytic activity was calculated as:

$$Q_{e} = \frac{{\left( {C_{0} - C} \right)}}{{C_{0} }}100\%$$

C_0_ was the starting concentration, and C was the final concentration at each time interval.

## Results and discussion

**Scheme 1:**
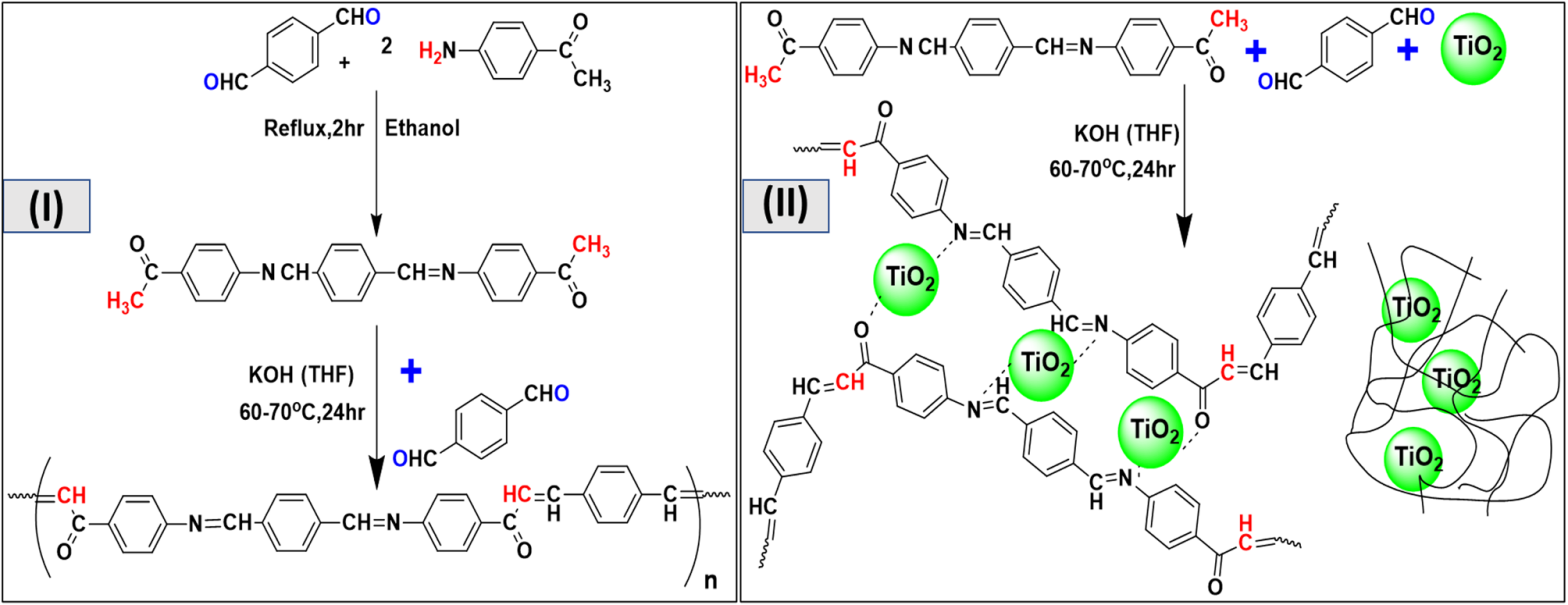
(I) preparation of monomer (CA) and polymer (CPAA). (II) preparation of nanocomposites (CPAA/TiO_2_).

As illustrated in Scheme 1II, a simple condensation process using 4- aminoacetophenone and terephthaldehyde produces Schiff base monomer (CA). The reaction is carried out in a solution of warmed ethanol without a catalyst, and the product is purified with hot ethanol to eliminate reactants^31^. As a result, a high yield of the monomer molecule (> 70%) is achieved, and ^1^H and ^13^C-NMR, FT-IR, and mass spectrometry characterize its formation.

**Figure 1:**
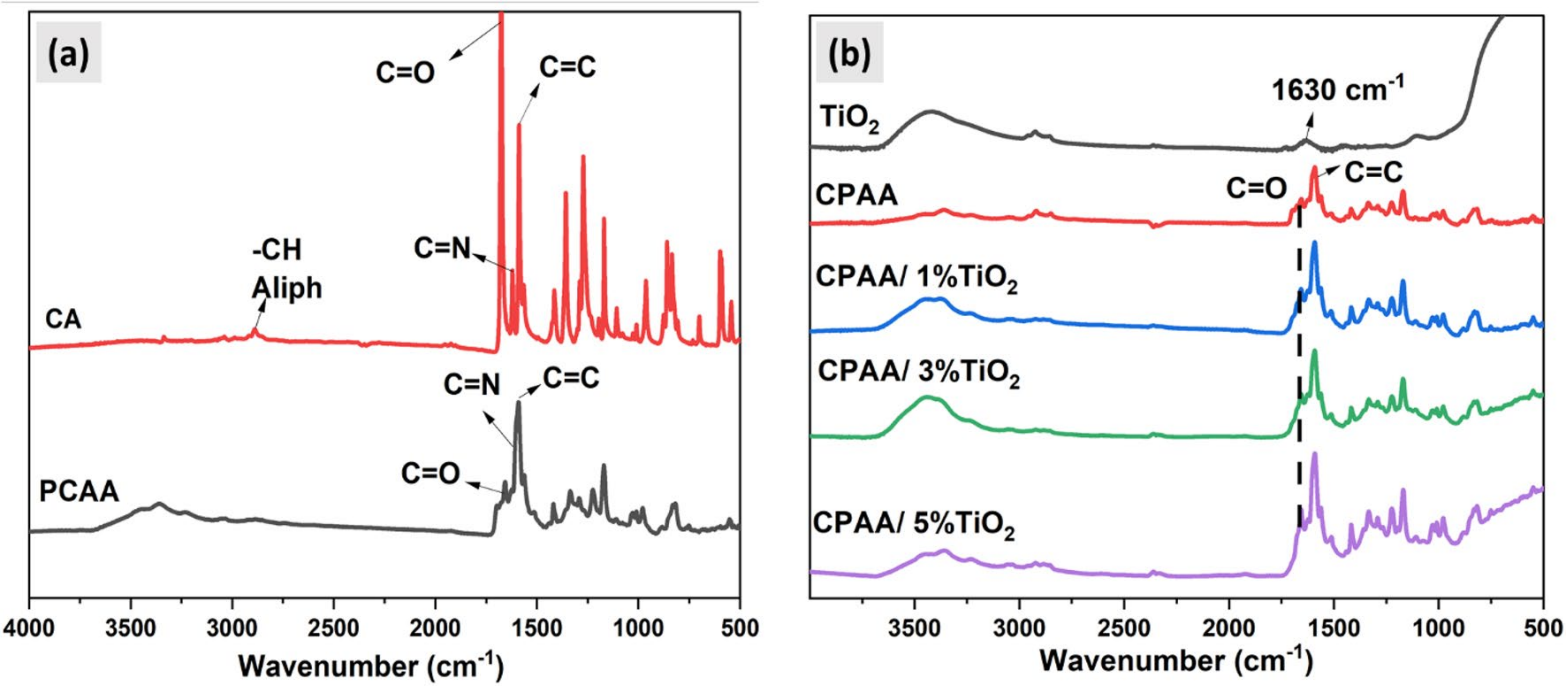
FT-IR spectra of (**a**) monomer (CA) and polymer (CPAA). (**b**) Nanocomposites (CPAA/TiO_2_).

The distinctive bands of CA in (CDCL_3_) in ^1^H-NMR spectrum Fig. S1 are observed at 8.19 ppm for CH=N and 8.05–7.33 ppm for benzene rings^32^. ^13^C-NMR for carbonyl groups of the acetophenone moiety (C=O) are demonstrated in Fig. S2 at 197.09 ppm, methyl carbon close to (C=O) at 26.51 ppm, carbon that condensed from terephthaldehyde (N=CH) at 160.6 ppm and signals at 155.89, 138.65, 134.99, 129.77, 129.45, and 120. The FT-IR spectrum of the azomethine monomer in Fig. 1a lacks the stretching bands of the reactants (–CHO 1700 cm^−1^ and –NH_2_ 3400 cm^−1^) and indicates a –CH=N– bond at 1620 cm^−1^. One distinctive feature is the location of the stretching band typical of the aliphatic methyl group of acetophenone at 2889 cm^−1^ and the alkene C=C bond at 1588 cm^−1^ in the FT-IR spectra. Additionally, the mass spectrum in Fig. S3 shows that a monomer molecule formed at m/z = 368.2, corresponding to its molecular weight.

According to Scheme 1II, CPAA was prepared by performing a condensation reaction under N_2_ between the produced monomer CA and terephthaldehyde in a basic medium employing THF as a solvent^33,34^. As a result, the chain of CPAA has the advantage of having double bonds conjugated to –C=N–, which is a highly desirable alternative to vinylene connections and allows for the display of semiconducting characteristics and various appealing applications^30,35^.

FT-IR spectroscopy supports the resulting CPAA structure; Fig. 1a displays the observed absorption bands in the resulting polymer and their assignments. There is an alteration to lower wavelengths compared to the monomer and the absence of the aliphatic peak CH_3_ group at 2889 cm^−1^ in the IR range of CPAA; the CH=N (imine) bond can be seen at 1609 cm^−1^, and the carbonyl carbon is at 1658 cm^−1^ indicating the polymer formation^32,36–38^. In addition, the existence of repeated monomer units causes a reduction in the wave number and intensity of the conjugated bonds in the polymer, leading to a semi-broad combined peak in the CPAA spectrum.

The nanocomposites CPAA/TiO_2_ are generated as CPAA polymer by the in-situ condensation polymerization method (Scheme 1II). Various weights of TiO_2_ nanoparticles (25 nm) are added as a percentage of the initial monomer and dispersed in the reaction solution during the formation of CPAA, then performing the same conditions to produce the nanocomposites. CPAA/TiO_2_ nanocomposites are investigated using (FT-IR) spectroscopy. As shown in Fig. 1b, the usual vibration bands for CPAA and TiO_2_ are similar and comparable to the original polymer. Broad bands at 3425 cm^−1^ and 1630 cm^−1^ are assigned to the stretching vibration of (–OH) and Ti–OH deformative vibration, respectively, (the moisture on TiO_2_ nanoparticles surface)^39^. The FT-IR spectra also reveal typical CPAA bond vibrations in the polymer's C=C and C=N bonds in the 1588–1596 cm^–1^ range and low-intensity peaks at 1413 cm^–1^ that correspond to =C–CH and –CH in- and out-of-plane deformation^36^. The C–N in-plane absorbance is 1043 cm^−1^, whereas the out-of-plane deformations are 1290 and 1170 cm^−1^, respectively. The interaction between TiO_2_ and CPAA is described by changes in certain characteristic peaks of CPAA, such as broadening and shifting vibrational peaks of OH groups on the TiO_2_ surface toward higher wave numbers and the intensification of the C=C bond of polymer. The interaction of OH groups on TiO_2_ surface with carbonyl group C=O of the polymer matrix causes a significant decrease in the absorption of (C=O) vibration present in the polymer backbone, and its position is transferred toward a lower wavenumber, and this is visible as the filler content increases.

**Figure 2:**
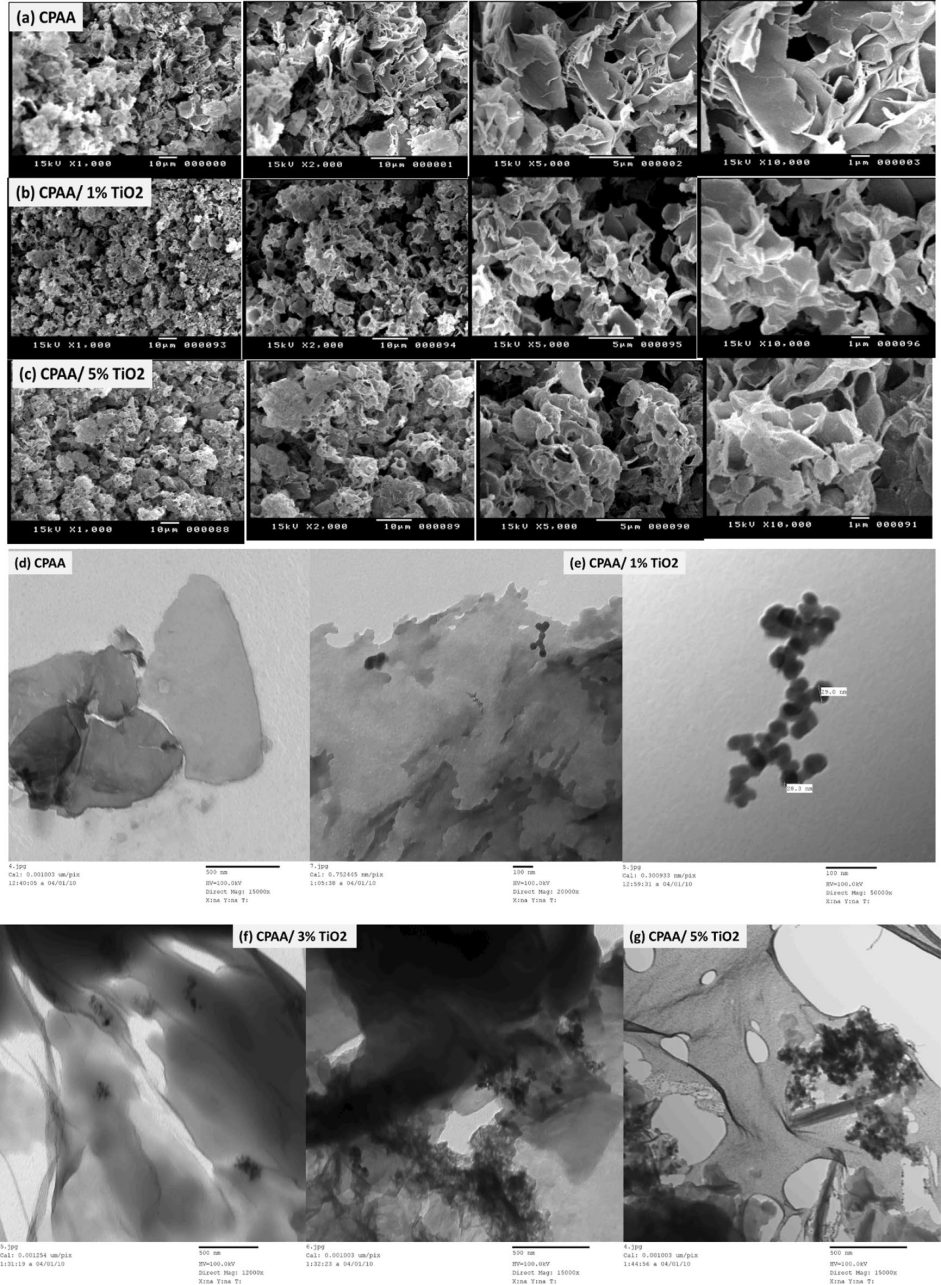
SEM images of (**a**) polymer (CPAA) and (**b**,**c**) nanocomposites (CPAA/TiO_2_ 1%, 5%). TEM images of (**d**) polymer (CPAA) and (**e**–**g**) nanocomposites (CPAA/TiO_2_ 1%, 3%, 5%).

SEM images in Fig. 2a–c show a closer look at the internal structure of the samples; CPAA and (CPAA/ TiO_2_ 1% and 5%) are chosen as the samples for the SEM study. The photos show clearly that the CPAA is uniform and homogeneous, with tightly connected aggregate layers in shape. At a higher magnification, the product comprises assembled layers with a cavity^40^. Nanocomposites exhibit a more uniform morphology and aggregation that has become more tightened due to the presence of particles with diameters of nanometers, which results in the removal of these cavities^41^. We can figure out that the amount of TiO_2_ in the nanocomposites greatly affects their morphology, revealing structural differences and indicating a reaction between CPAA and TiO_2,_ especially at a higher concentration of 5% TiO_2_. A TEM examination was done to validate the dispersion of TiO_2_ particles inside the polymer, as illustrated in Fig. 2d–g. Nanocomposites' TEM pictures show nanoparticles decorated and aggregated across the polymer surface; these nanoparticles have a spherical shae with typical sizes of around 25–29 nm and a reasonably narrow size dispersion. The growing amount of nanoparticles embedded inside the polymer surface as the nanoparticle concentration rises indicates effective nanoparticle incorporation into the polymer matrix^42^.

**Figure 3:**
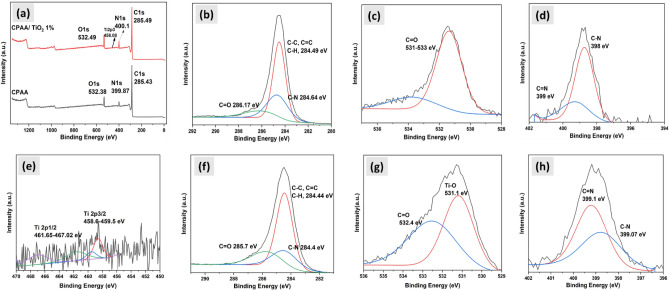
XPS spectra of (**a**) CPAA and CPAA/TiO_2_ 1%, (**b**) C 1 s, (**c**) O 1 s, (**d**) N 1 s of CPAA and (**e**) Ti 2p (**f**) C 1 s, (**g**) O 1 s, (**h**) N 1 s of CPAA/TiO_2_ 1%.

Surface chemical components of CPAA and its nanocomposite were characterized using X-ray photoelectron spectroscopy (XPS) (Fig. 3a–h). In Fig. 3b, we observe that the signals corresponding to the C 1 s of CPAA exhibit peaks from C–C (284.49 eV), C–N (284.64 eV), and C=O (286.17 eV). The signals at 531–533 eV in Fig. 3c represent the C=O of O 1 s. The C=N– of the polymer structure is located at 399 eV, whereas the C-N bond is related to the signal at 398.0 eV, as shown in Fig. 3d. The elements C, O, Ti, and N can be identified in the XPS spectrum of CPAA/TiO_2_ 1% (Fig. 3a), with binding energies of 285.49, 532.49, 458.08, and 400.1 eV, respectively. XPS spectra of the Ti 2p area are shown in Fig. 3e. TiO_2_ nanoparticles have a Ti 2p spectrum corresponding to a binding energy of 458.1 eV for Ti 2p3/2 and 467.1 eV for Ti 2p1/2. Binding energies of 284.4, 284.44, and 285.7 eV are predicted for C1s, while those of 531 and 532 eV are predicted for O1s, and those of 399.07 and 399.1 eV are predicted for N1s based on the data. The proportions of carbon, oxygen, and nitrogen in CPAA were 88.24%, 7.57%, and 4.19%, respectively, whereas CPAA/TiO_2_ 1% contained 84.85% carbon, 9.42% oxygen, 0.12% titanium, and 5.61% nitrogen in composition. All of these findings show that an excellent combination of TiO_2_ and CPAA^42,43^.

**Figure 4:**
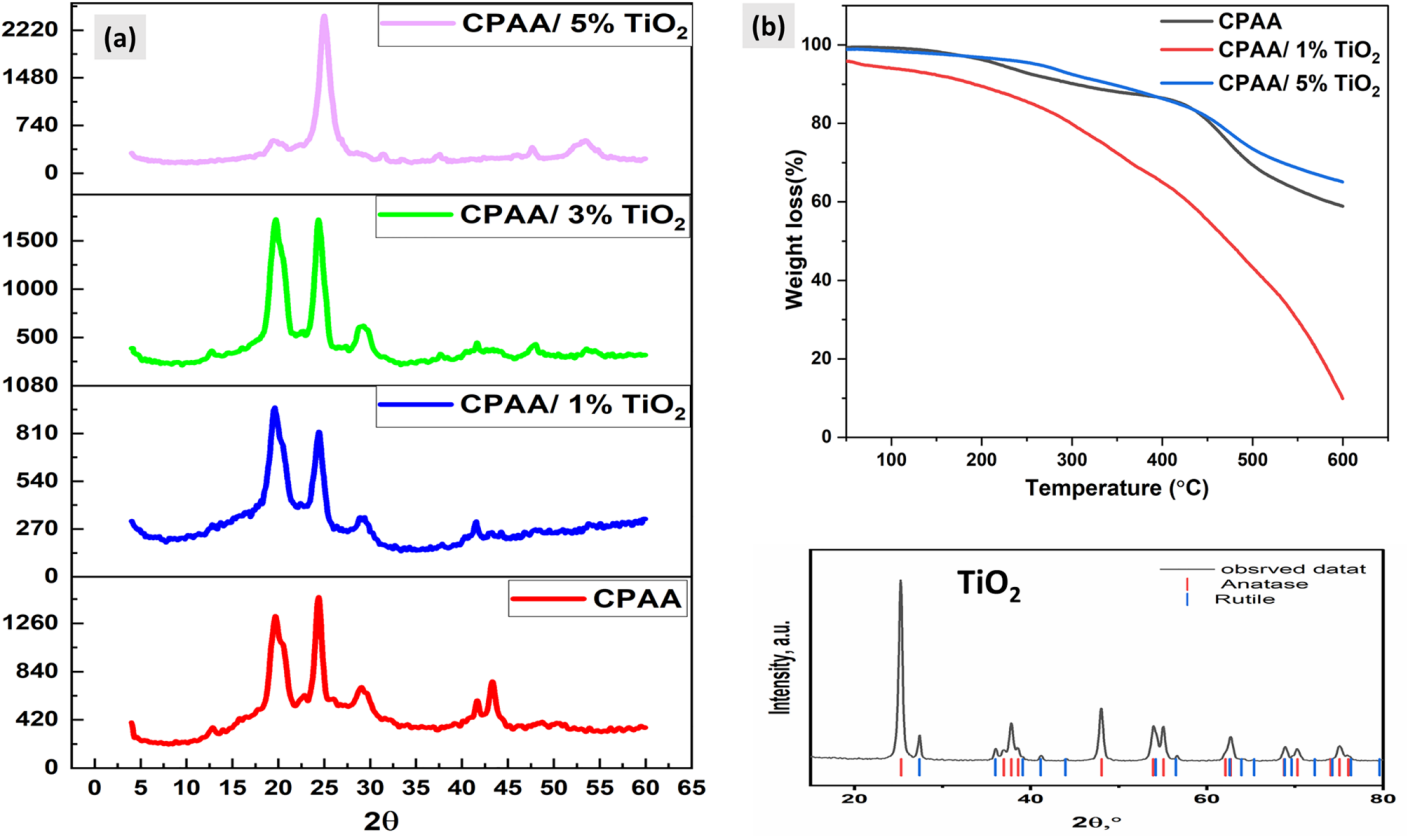
(**a**) X-RD pattern of TiO_2_, polymer (CPAA), and nanocomposites (CPAA/TiO_2_). (**b**) TGA graphs of polymer (CPAA) and nanocomposites (CPAA/TiO_2_ 1%, 5%).

The crystalline characteristics of CPAA and CPAA/TiO_2_ nanocomposites were examined using X-ray powder diffraction. According to Fig. 4a, the semicrystalline nature of CPAA results from the orientation and structure of its unit structure. The conjugated structure C=C–C gives it its conductive properties because the electrons in that bond are free to flow along the molecule chain as they are in metals. Additionally, electrons can move between chains that are next to one another. To facilitate the transmission of electrons, a molecule chain's length, number, and regularity of arrangement all increase as it grows longer^44^. The observed XRD pattern of pure TiO_2_ confirmed that the material has two phases anatase and rutile. The distinctive peaks of TiO_2_ can be seen in the CPAA/TiO_2_ hybrids. The distribution and concentration influence of TiO_2_ nanoparticles affects the intensity of CPAA-related peaks, as seen in the XRD pattern. Peak intensity rises with rising TiO_2_ percentage in the composite, suggesting complete interaction between the CPAA and TiO_2_^45,46^.

Figure 4b depicts the results of a thermal degradation analysis using TGA on CPAA and its TiO_2_ nanocomposites in the N_2_ environment. This analysis provides information on the structural stability of the polymer and the in situ-produced nanocomposites. It is evident from the data in Table 1 and the TGA graphs that CPAA and its nanocomposites undergo weight loss in three successive steps. Adsorbed moisture and solvents are lost at temperatures between 25 and 140 °C, leading to the initial breakdown. The second decrease in mass between 210 and 400 °C is due to removing unreacted monomers and oligomers. The third significant weight loss occurs around 430 °C, and it is due to the polymer backbone breaking apart. Thermal degradation of the polymer nanocomposite with 5% TiO_2_ is much lower than that of pure CPAA, as seen in the graph^47^. The thermal stability of 5% TiO_2_ suggests that this percent of nanoparticle improves the polymer's heat resistance, mostly attributable to increased interfacial contact between the polymer and nanoparticles, as demonstrated by the previous SEM pictures. There is also a definite relationship between composite morphologies and thermal characteristics. The thermal decomposition of the nanocomposite 1% TiO_2_ begins at a significantly lower temperature than the thermal decomposition of the polymer, and the degradation occurs rapidly. However, the nanocomposite containing 1% sample exhibits a nearly distinct decomposition pattern over the entire region compared to the polymer. This behaviour can be attributed to the difference in the distribution of TiO_2_ nanoparticles in the polymer matrix and the strong coordination contact between CPAA and TiO_2_, which likely decreased the interchain connections in CPAA and assisted the polymer's heat breakdown^48,49^. The SEM pictures have already established that the composite containing 1% nanoparticles displays a homogenous nanoparticle distribution. The interparticle distances are too short at increasing nanoparticle concentrations, and the particles overlap to form tiny aggregates in the CPAA matrix. This results in a difference between the thermal stability of the 5% composite and the 1% TiO_2_ samples. The final residue for 1 and 5% TiO_2_ of composites at 600 °C is 10 and 65%, respectively. The char residue increase indicates the nanocomposite's thermal resistance, and this char layer acts as a protective covering on the surface of the composite material, preventing further combustion.

**Table 1:** Temperature (°C) for various decomposition levels in N_2_ at a heating rate of 10 °C/min.

Sample	10% wt. Loss	20% wt. Loss	30% wt. Loss	40% wt. Loss	50% wt. Loss	Char Yield (%) at 600 °C
CPAA	303	454	497	585	> 600	59
CPAA/1% TiO_2_	191	299	365	429	473	10
CPAA/5% TiO_2_	344	461	533	> 600	> 600	65

**Figure 5:**
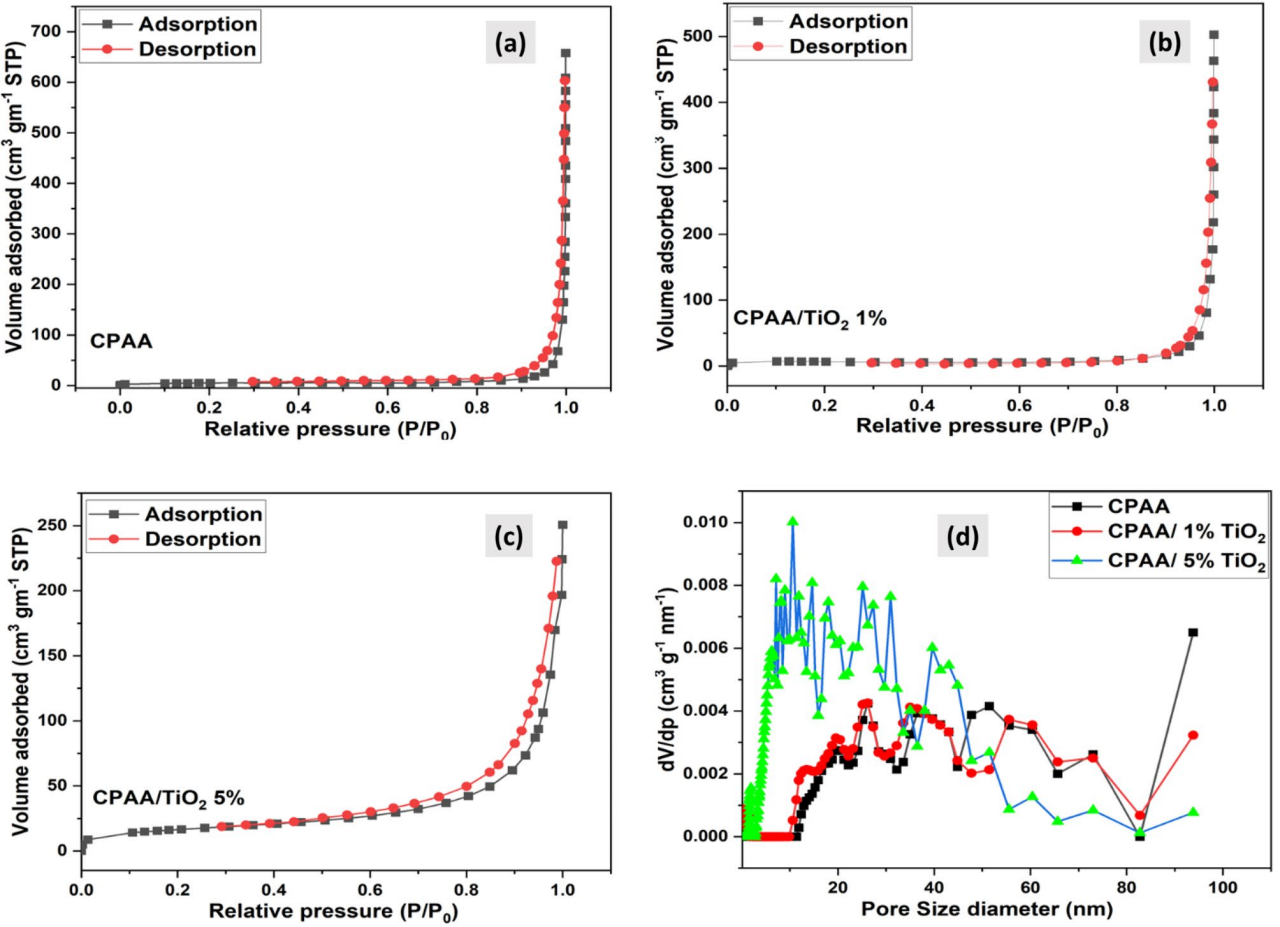
(**a**–**c**) BET surface area analysis, (**d**) pore size distribution of (CPAA and nanocomposites CPAA/ TiO_2_ 1%, 5%).

N_2_ adsorption was used to determine the nanocomposites' BET surface area and porous structure Fig. 5a–d. After TiO_2_ NPs inclusion, the BET surface area of the mesoporous CPAA increased from 17.87 m^2^ g^−1^ to (27.62 m^2^ g^−1^ and 58.33 m^2^ g^−1^) in the nanocomposites CPAA/TiO_2_ 1% and CPAA/TiO_2_ 5%, respectively. The pore diameter of CPAA 42.12 nm was seen to be reduced to 27.72 nm and 19.27 nm in the CPAA/TiO_2_ 1% and CPAA/TiO_2_ 5% nanocomposites, respectively. Unlike nanocomposites, the polymer has a large pore size and a low BET surface area. The increased surface area of nanocomposites may result from the uniform penetration of TiO_2_ NPs into the polymer chains.

**Figure 6:**
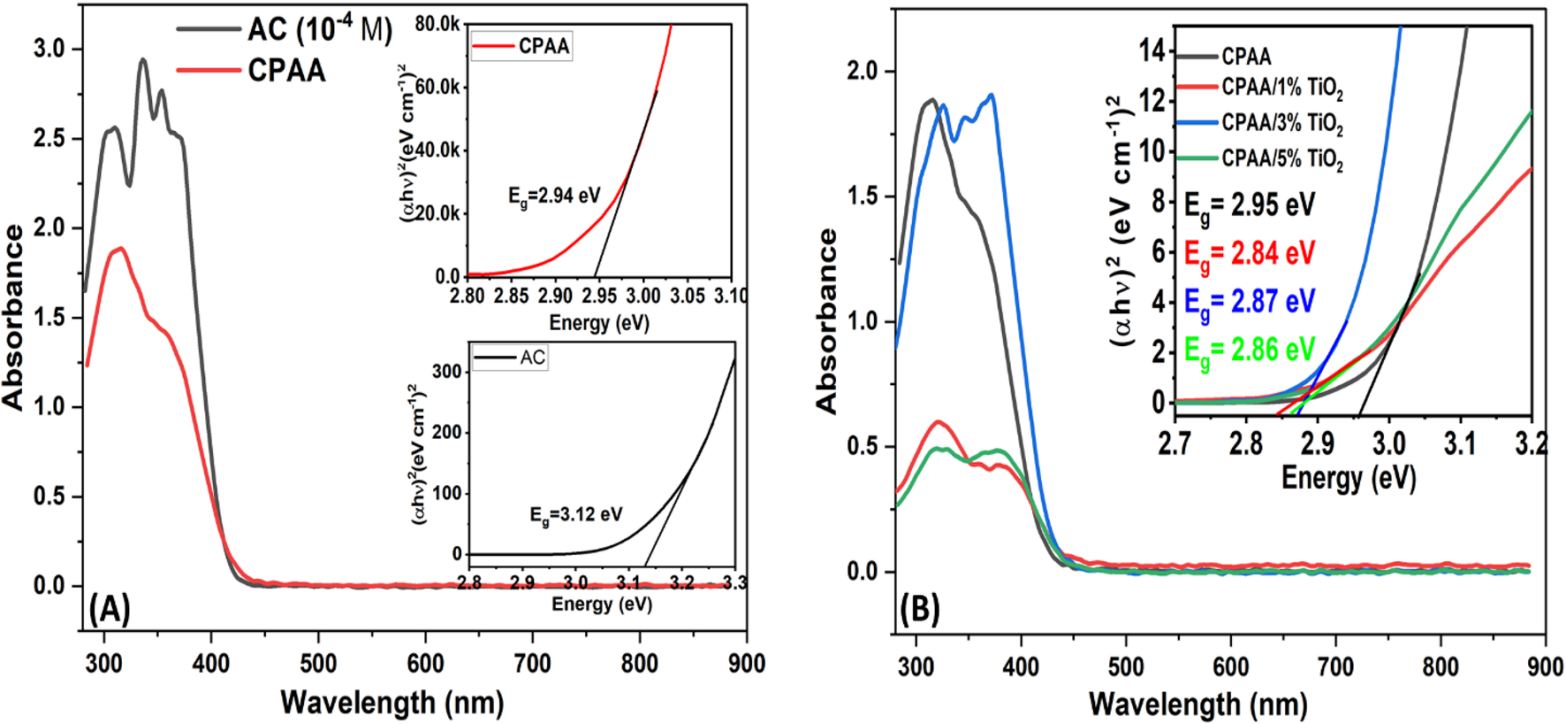
UV- Visible spectra of polymer (CPAA) and nanocomposites (CPAA/TiO_2_ 1%,3% 5%). Inset graphs are the energy band gap from the Tauc equation of samples.

UV–visible absorption is used to identify the light absorption properties of AC, CPAA, and nanocomposites. Band gap energy was determined using Tauc's equation (by extrapolating the linear portions of the curves), and the UV–visible absorption spectra are shown in Fig. 6. The measurements are carried out in DMF solvent, with 10^–4^ CA solution, 0.01 mg in 10 ml of CPAA, and 1 mg in 10 ml for nanocomposites. The CA monomer molecule exhibits a maximum of three absorption bands at 308 nm for (n–π*) of C=N; and bands with shoulders at 336 nm, 356 nm, and 372 nm for (n–π*) of C=O and (π–π*) transition of benzenoid rings. It appears to have a band gap energy of approximately 3.12 eV due to the (n–π*) transition and the (π–π*) transition of the benzene ring. The polymer exhibits a peak with a red shift and shoulder for both (n–π*) transition and (π–π *) transition of aromatic around (314 nm, 356 nm) and band gap (Eg = 2.95 eV), which could be attributed to the wide distribution of conjugated chain length in conducting polymers. The spectra of nanocomposites show that the absorption occurs in the same regions as the pure polymer but with a red shift and a different intensity. The E_g_ values of the resulting nanocomposites are changed and decreased from 2.95 eV to 2.84 eV, 2.86 eV, and 2.87 eV. This shows that the polymer and TiO_2_ nanoparticles react in a way that makes the polymer more semiconducting.

### Photocatalysis mechanism

The photocatalysis process generally consists of three main steps: photocatalyst absorbs the light, separation of photogenerated charge carriers, and interfacial catalytic redox reactions. During the UV–vis light exposure of the sample, a photon excites from the valence band VB to the conduction band CB according to π-π* electronic transition. This excited electron roams to the sample surface and initiates the adsorbed oxygen molecules to make superoxide radicals (O^2**.**−^), which can oxidize organic impurities in the solution system. In the meantime, the holes produced from photogeneration can also oxidize organics straight. The general process can be described as follows.

$${\text{Polymer sample }} + {\text{ photon energy }} \to {\text{ Polymer sample}}(e^{ - }_{LUMO} \ldots .h^{ + }_{HOMO} )$$

$$\begin{gathered} e^{ - } + O_{2} \to O_{2}^{. - } \hfill \\ O_{2}^{. - } + H^{ + } \to .OOH \hfill \\ 2.OOH \to O_{2} + H_{2} O_{2} \hfill \\ H_{2} O_{2} + O_{2}^{. - } \to .OH + OH^{ - } + O_{2} \hfill \\ H_{2} O_{2} + h^{ + } \to 2OH \hfill \\ \end{gathered}$$

Demethylation of MB takes place when these reactive and non-selective radicals contact with MB dye, resulting in the breaking of the nitrogen-methyl link. After this, the aromatic ring is attacked by the radical species. The breakdown of the MB's aromatic ring is the primary source of the reaction intermediates. Eventually, the dye fragments are transformed into water, carbon dioxide, ammonium ions, and sulfate ions after passing through other chemical intermediates such as aldehyde, carboxylic species, phenols, and amines^29,50^.

**Figure 7:**
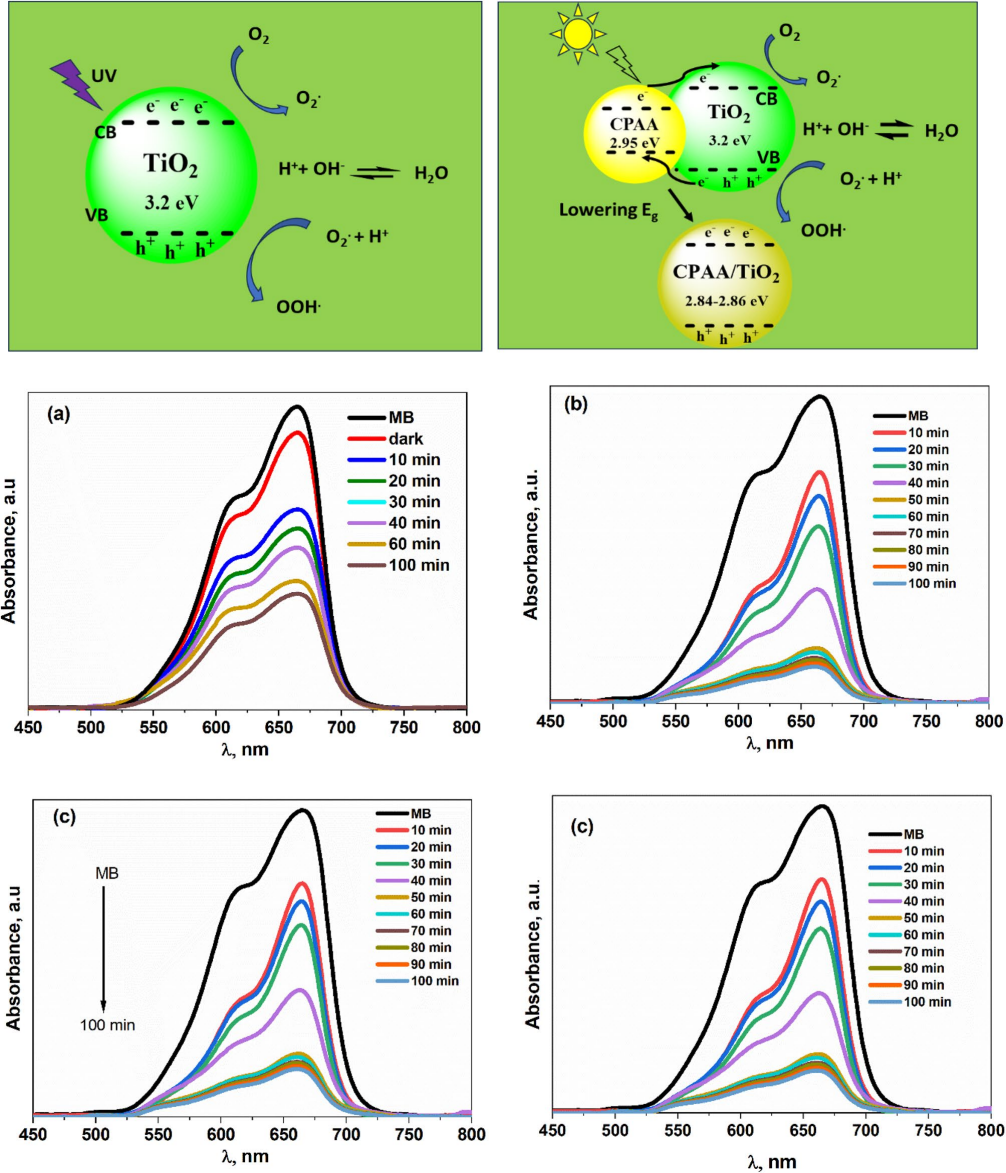
Photocatalytic mechanism of nanocomposites. UV–vis absorption spectrum of CPAA against various irradiation times under light irradiation of MB (**a**) 1%TiO_2_ (**b**) 3%TiO_2_ (**c**) and 5% TiO_2_ (**d**).

By exposing TiO_2_ nanoparticles to UV radiation, electron–hole pairs are created, which, when combined with water, produce hydroxyl and super-oxide radicals that may oxidize and break down organic and inorganic compounds. As a result of its 3.2 eV band gap, however, TiO_2_ nanoparticles can only be excited by UV light, limiting their ability to create electron–hole pairs. As a result of the low energy of UV light in solar photons (only 3–5%), poor photocatalytic efficiency is a problem when using solar energy. One way to address this deficiency is to boost TiO_2_'s photocatalytic efficacy by adding a narrow-band gap molecule as a sensitizer^51^. Because of the lower bandgap of CPAA than metal oxide, it functions as a photosensitizer in the CPAA-TiO_2_ hybrid, absorbing a wide range of visible light. The excited electrons at LUMO of CPAA chains are injected into the conduction band CB of a transition metal oxide (such as TiO_2_), which combines with an adsorbed water molecule to form radicals, whereas holes may react with water to form ^**.**^OH. Figure 7 depicts the photocatalytic mechanism of CPAA hybrids. Identifying actively engaged oxidative species in photocatalysis is critical for outlining reaction pathways. Radical scavengers that can specifically capture the target radicals are utilized to examine the involvement of the major oxidative species in photocatalysis.

### Photodegradation activity

**Figure 8:**
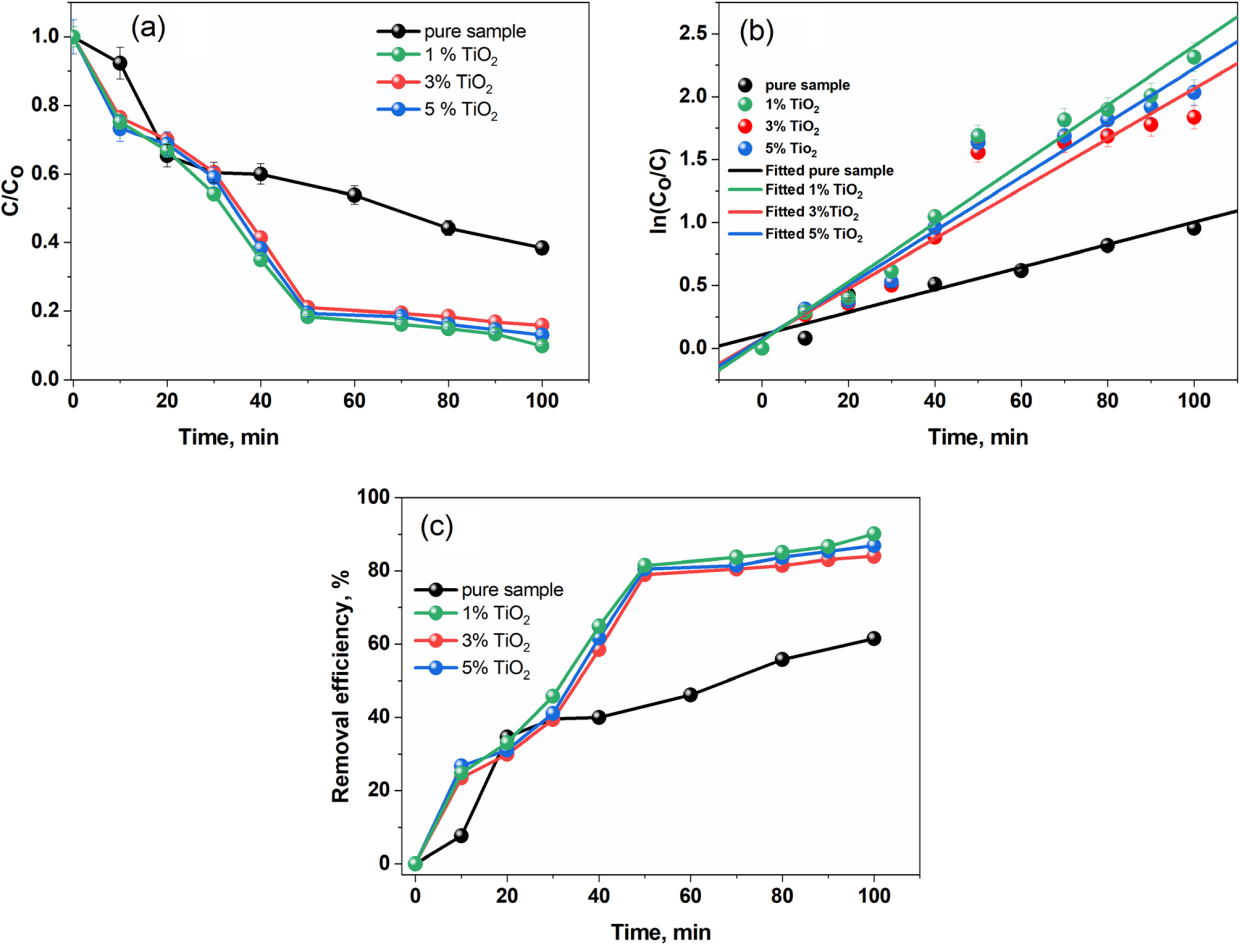
(**a**) C/C_o_ plots of MB dye concerning time over CPAA and doped with different concentrations of TiO_2_ under sunlight. (**b**) Comparison of apparent rate constants of CPAA and doped with different concentrations of TiO_2_ towards MB dye under natural sunlight irradiation. (**c**) Removal efficiency of a CPAA doped with different concentrations of TiO_2_ under sunlight.

The photocatalytic performance of the pure sample and doped with TiO_2_ against MB dye were estimated under UV–Vis light irradiates and dark. In the beginning, the photodegradation performance of the MB dye was carried out in the photocatalyst absence (pure-CPAA, CPAA-TiO_2_ (1–5)%) under dark requirements, which exhibits no decomposition after 200 min. Figure 8b displays the kinetics charts resulting from first-order linear transform:

$$- \ln \left( {\frac{C}{{C_{0} }}} \right) = Kt$$

Nevertheless, the efficiency of the degradation of pure-CPAA, CPAA- TiO_2_ (1–5)% against MB dye under dark conditions is 15, 22, 28, and 34.5%. These results illustrate that the (pure-CPAA, CPAA- TiO_2_ (1–5)%) photocatalysts were necessary for photocatalytic performance. On the one hand, under sunlight, the degradation efficiency of CPAA over MB dyes is 62%, as shown in Fig. 8c and Table 2. Similarly, CPAA-TiO_2_ 1% destroyed 90% of MB dyes under simulated sunlight irradiation. It was discovered that the low band gap of CPAA@TiO_2_ 1% (2.84 eV) accelerates high electron–hole recombination, increasing photocatalytic activity. On the other hand, the polymer doped with 3% TiO_2_ demonstrates the ability of degradation for MB was 84% according to its energy gap at around 2.87 eV. However, the increase in TiO_2_ to 5% in the CPAA led to an enhancement of the removal efficiency to 86%, as seen in Fig. 8c.

**Table 2:** Degradation % and linear fitting parameters.

Catalyst	Dye	Slope	R^2^	Energy gap (eV)	Degredation%
Pure CPAA	MB	0.00896	0.89792	2.95	62
1% TiO_2_		0.0199	0.9095	2.84	90
3% TiO_2_		0.02152	0.92597	2.87	84
5%TiO_2_		0.02345	0.94614	2.86	87

The kind of material being used is the single most important aspect in determining how quickly the MB degrades; depending on the material, the conjugation in the polymer may assist lower the band gap and speed up the degradation process. It is always necessary to modify polymers to make them more efficient. Modified materials have more complicated structural and morphological features, and they have been shown to degrade faster than virgin polymers. The alteration may be achieved by hybridizing with another polymer or nanoparticles, resulting in composites or nanocomposites, respectively. Additionally, the effectiveness of the nanocomposites for MB degradation is greatly affected by the morphology of the nanoparticles. According to the analysis results, all of the prepared nanocomposites' characteristics vary from those of the original polymer, with the most significant features having a considerable impact on the efficiency of their morphology and surface area. When compared to the original polymer, the resultant nanocomposites with increased surface area and roughness or other complex morphologies features show improved photocatalytic performance^52^. The dye's photocatalytic rate has been greatly affected by the concentration of nanoparticles introduced to the reaction media. Initially, it was observed that the degradation rate of MB increased as a function of the concentration of nanoparticles in the medium. However, the rate begins to decrease after reaching the particular concentration known as the optimal concentration. One possible explanation for the first rise is that, when nanoparticle concentrations rise, there are more interaction regions of nucleation for the MB dye, which leads to an initial increase. As a result of free radicals produced by the nucleation sites during irradiation treatment, the dye degradation process is accelerated. Once the optimal concentration of nanoparticles is reached, the photocatalyst's efficiency drops with each successive increment. When there are too many nanoparticles in the reaction media, their effectiveness drops. As a result of the continual collisions caused by overpopulation and the effectiveness with which nanoparticles function, aggregation occurs. Light scattering becomes the dominant phenomenon in the reaction media, and nanoparticle aggregation causes the solution to become turbid^53^. After reaching the optimal concentration, this component also decreases the nanocomposites' effectiveness since light is now dispersed instead of used in the activation process^54^.

The results show that adding TiO_2_ in the CPAA matrix was crucial in the degradation of MB dye under light exposure. The polymer matrix stabilized the TiO_2_ nanoparticles and reduced the recombination of photogenerated electron–hole pairs, facilitating interfacial charge transfer between CPAA and TiO_2_. Furthermore, the polymer@ TiO_2_ 1% has a larger surface area and pore width than the pure CPAA, which is an essential element in determining degrading efficiency, as shown in Fig. 8a–c.

**Figure 9:**
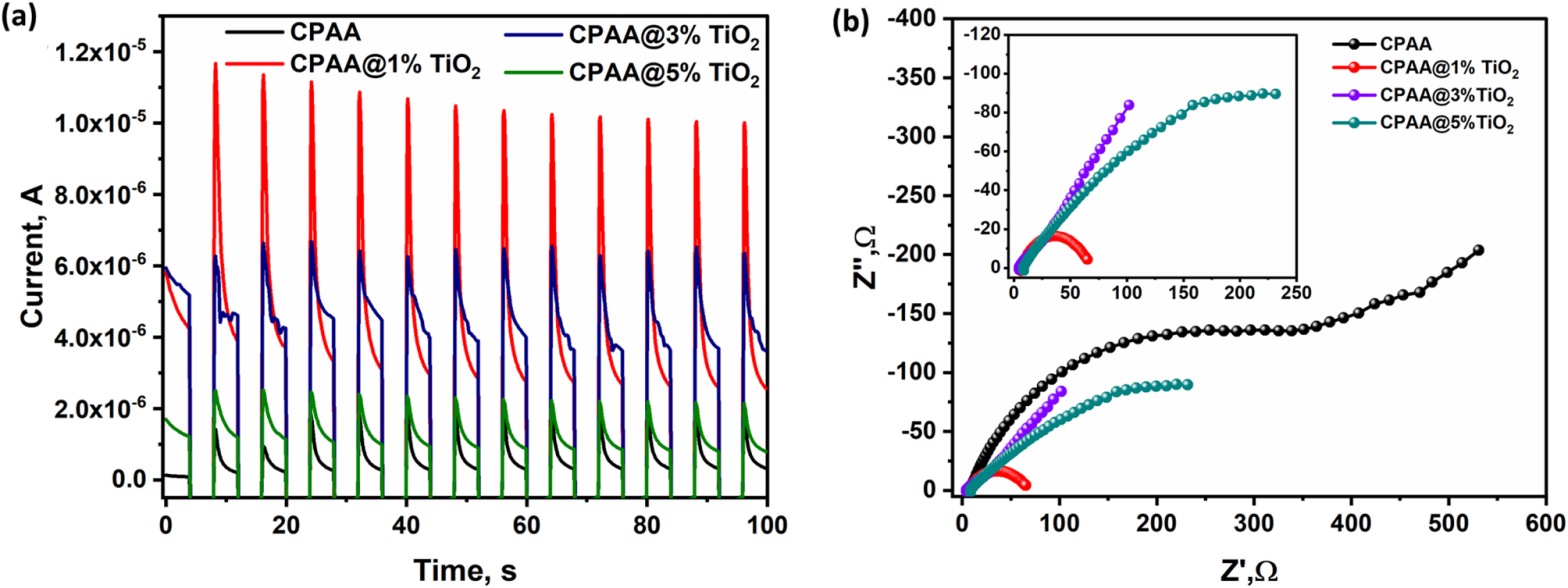
(**a**) Photocurrent (I-t) behaviors of both pure CPAA and electrodes containing 1, 3 and 5%TiO_2_ under visible light exposure. (**b**) EIS plots of CPAA and its nanocomposites.

Figure 9a displays the transient photocurrent (I-t) behaviors of both pure CPAA and electrodes containing 1, 3, and 5%TiO_2_ under visible light exposure. This data can be utilized to assess the effectiveness of charge generation and separation. As depicted, the photocurrent generated by the 1.0% CPAA@1% TiO_2_ electrode is four times as much as that produced by the pure CPAA electrode. This increase in the photocurrent of CPAA refers to the insertion of TiO_2_, indicating that photogenerated electrons and holes were disconnected more efficiently. The ability of electron transfer among various electrodes was additionally examined using electrochemical impedance spectroscopy (EIS), with the corresponding spectra presented in Fig. 9b. A significant decrease in resistance observed in the CPAA@1%TiO2 electrodes suggests that the incorporation of TiO_2_ has notably diminished the charge-transfer resistance. This enhancement facilitates the more efficient separation of photogenerated electron–hole pairs, as shown in a set of Fig. 9b.

**Figure 10:**
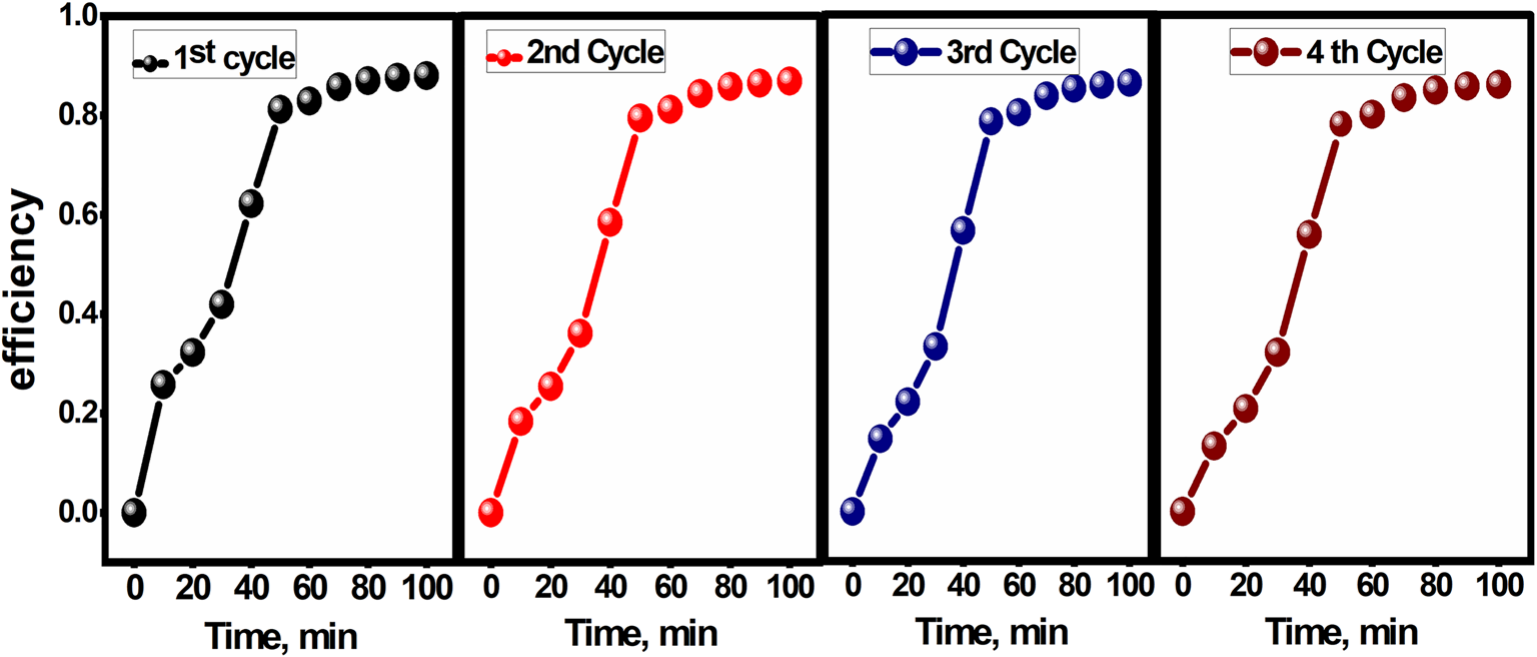
Photocatalytic recycle degradation of MB as a function of the irradiation period over the CPAA@1% TiO_2_.

**Figure 11:**
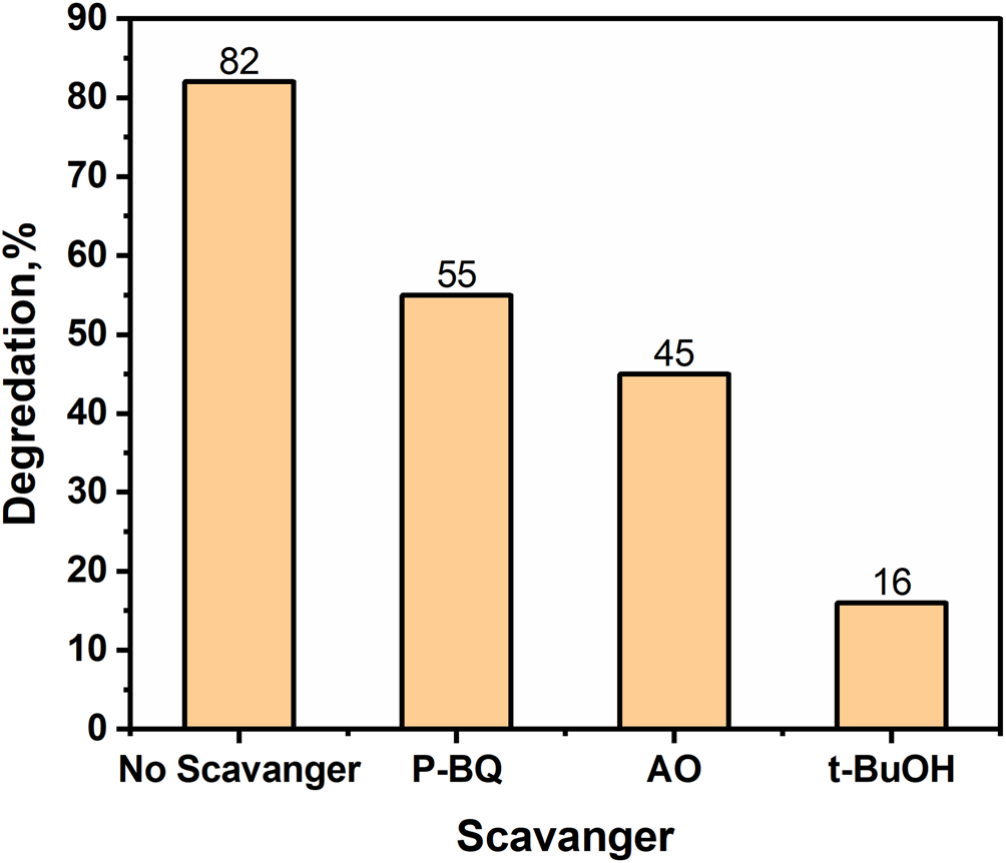
The influence of numerous scavengers on the degradation of photocatalysis of MB.

The recyclability of the photocatalysts plays a crucial role in their practical usage. Figure 10 demonstrates the recyclability of the CPAA@1%TiO_2_ composite. Even after undergoing four consecutive cycles, the composite retains approximately 90% degradation rate of MB after around 100 min of VL irradiation, highlighting its excellent recyclability. Figure 11 illustrates the percentage of photocatalytic degradation of MB dye with and without the presence of scavengers. While the degradation of MB dye reached 82% in the absence of any quencher, it decreased to 55% and 45% in the presence of P-BQ and AO, respectively. Nonetheless, the degradation of MB notably dropped to 16% with t-BuoH, indicating that electrons are predominantly accountable for the photodegradation of MB dye via the generation of ˙O_2_^−^ radicals.

## Conclusion

The pure CPAA and CPAA/TiO_2_ nanocomposite were prepared via the in-situ technique. The spectrum of FT-IR showed well-agreed peaks, evidenced by the nanocomposite formation. The materials are suitable for the photodegradation of dye due to the band gap ranging from 2.95 to 2.84 eV under simulated sunlight. The SEM clearly shows that the CPAA is uniform and homogeneous, with tightly connected aggregate layers in shape. However, the amount of TiO_2_ in the nanocomposites greatly affects their morphology, revealing structural differences and indicating a reaction between CPAA and TiO_2_, especially at a higher concentration of 5% TiO_2_. The removal efficiency of pure-CPAA over MB dye under simulated sunlight was 62%; however, 1% TiO_2_ doped with CPAA destroyed 90% of MB dyes. It was discovered that the low band gap of CPAA@1%TiO_2_ (2.84 eV) accelerates high electron–hole recombination, increasing photocatalytic activity. On the other hand, the polymer doped with 3% TiO_2_ demonstrates the ability of degradation for MB was 84% according to its energy gap at around 2.87 eV. However, the increase in TiO_2_ to 5% in the CPAA led to an enhancement of the removal efficiency to 86%. The enhancement of CPAA performance is attributed to the unique optical, high surface area, particle uniformity, and surface properties. The photocatalytic activity follows a pseudo-first-order kinetic model. Therefore, the synthesized nanocomposite of the CPAA/TiO_2_ was an effective photocatalyst for wastewater remediation and other industrial applications.

### Supplementary Information

Supplementary Figures.
