# Author Correction: The enhanced photocatalytic performance of CPAA doping with different concentrations of Titanium oxide nanocomposite against MB dyes under simulated sunlight irradiations

**DOI:** 10.1038/s41598-024-66984-0

**Published:** 2024-07-12

**Authors:** Marwa M. Sayed, Abdelaziz M. Aboraia, Yara A. Kasem, Nancy N. Elewa, Yasser A. M. Ismail, Kamal I. Aly

**Affiliations:** 1 Chemistry Department, Faculty of Science, New Valley University, El-Kharja, 72511 Egypt; 2 Physics Department, Faculty of Science, Al-Azhar University, Assiut, 71542 Egypt; 3 Energy Storage Research Laboratory (ESRL), Physics Department, Faculty of Science, Al-Azhar University, Assiut, 71542 Egypt; 4 Polymer Research Laboratory, Chemistry Department, Faculty of Science, Assiut University, Assiut, 71516 Egypt; 5 Physics Department, Faculty of Science, Ain Shams University, Cairo, 11566 Egypt; 6 Department of Physics, Faculty of Science, Islamic University of Madinah, Madinah, Saudi Arabia

Correction to: *Scientific Reports* 10.1038/s41598-024-61983-7, published online 04 June 2024

The original version of this Article contained an error in the spelling of the author Nancy N. Elewa which was incorrectly given as Nancy N. Elew.

The original Article has been corrected.

