# Supplementary figures and images for: The enhanced photocatalytic performance of CPAA doping with different concentrations of Titanium oxide nanocomposite against MB dyes under simulated sunlight irradiations
Source: Sci Rep. 2024 Jun 4;14:12768. doi: 10.1038/s41598-024-61983-7 (PMC11150388; doi:10.1038/s41598-024-61983-7)

**
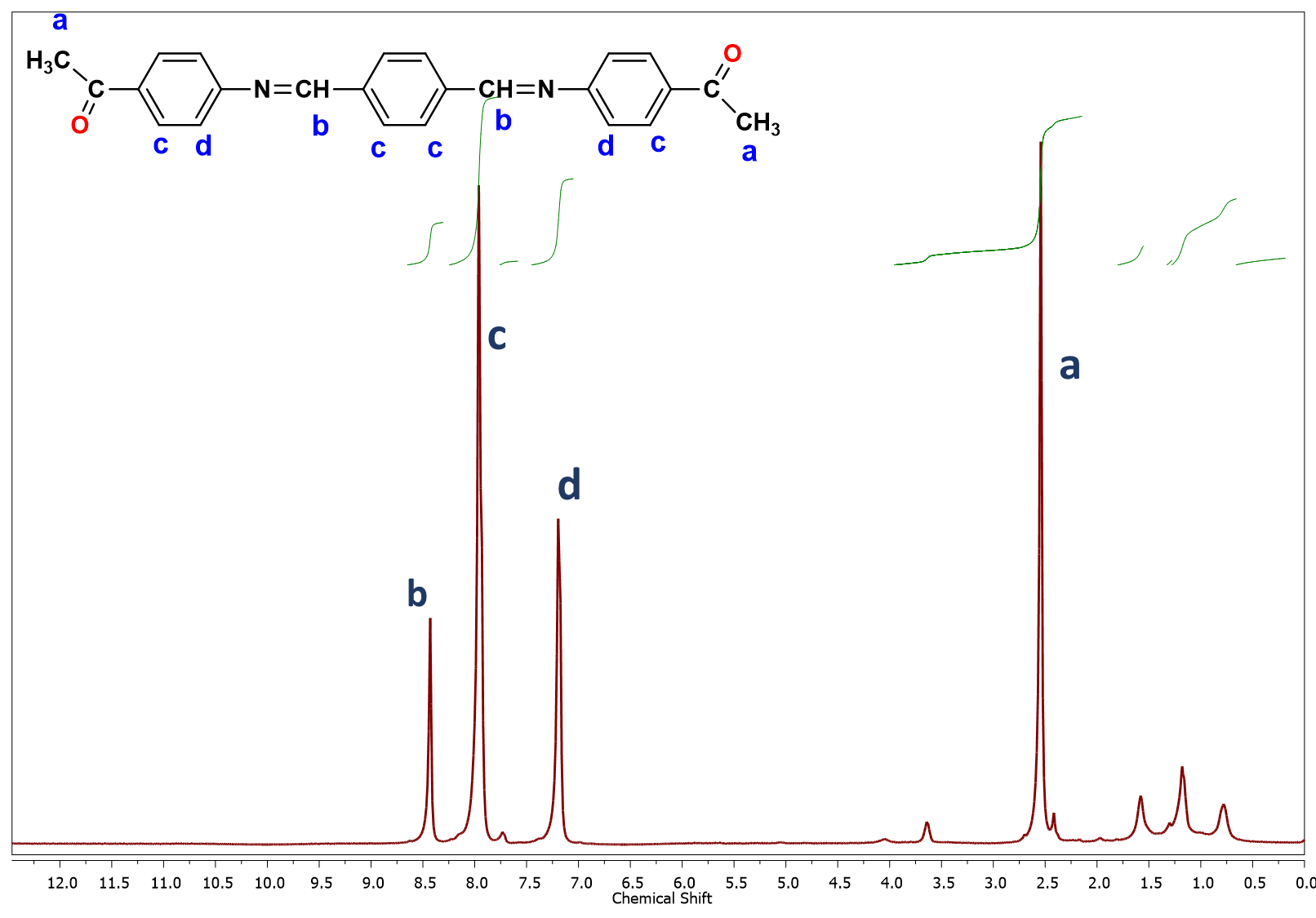
**

**Figure S1.** 1H-NMR spectrum of CA.


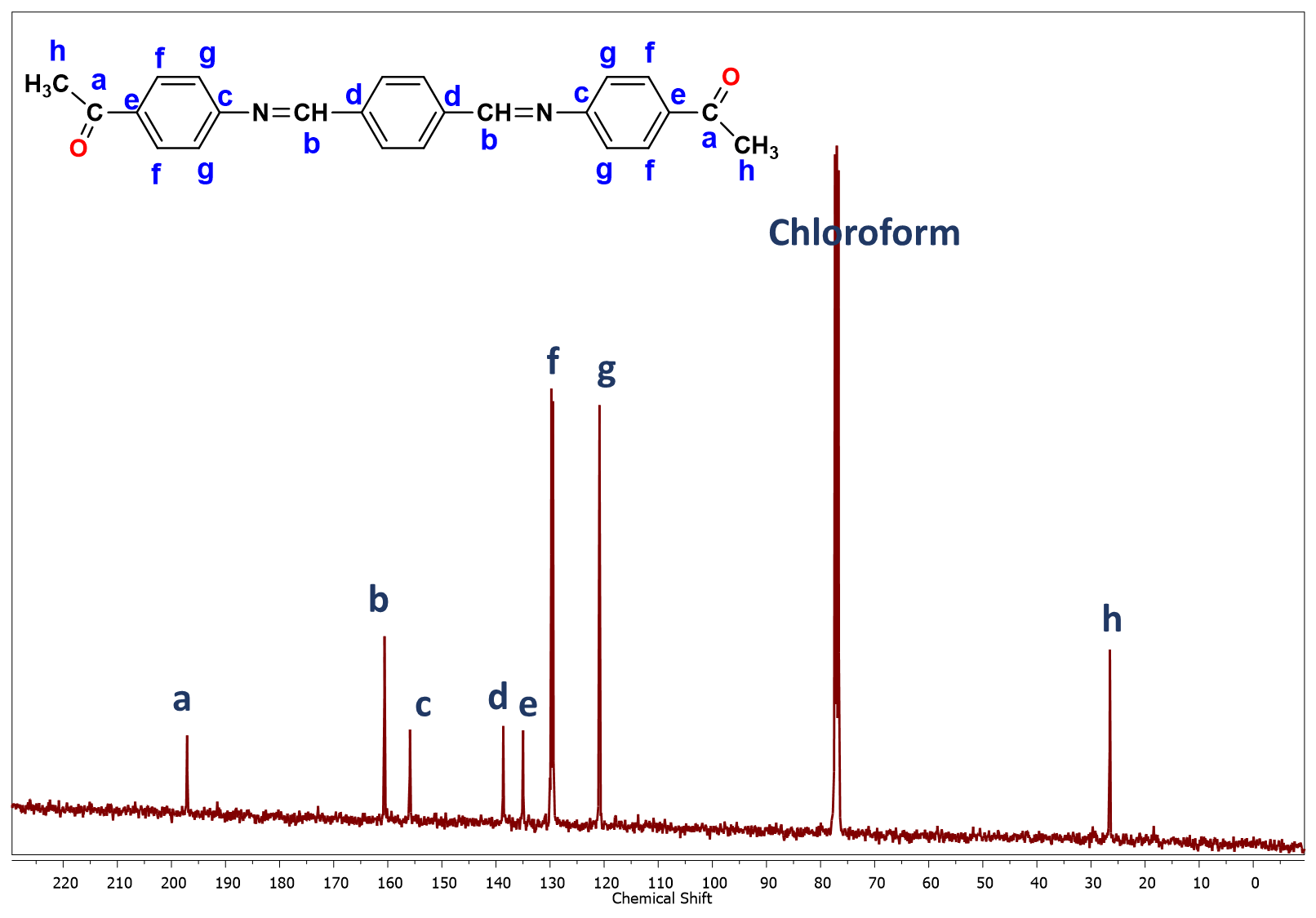


**Figure S2.** 13C-NMR spectrum of CA.


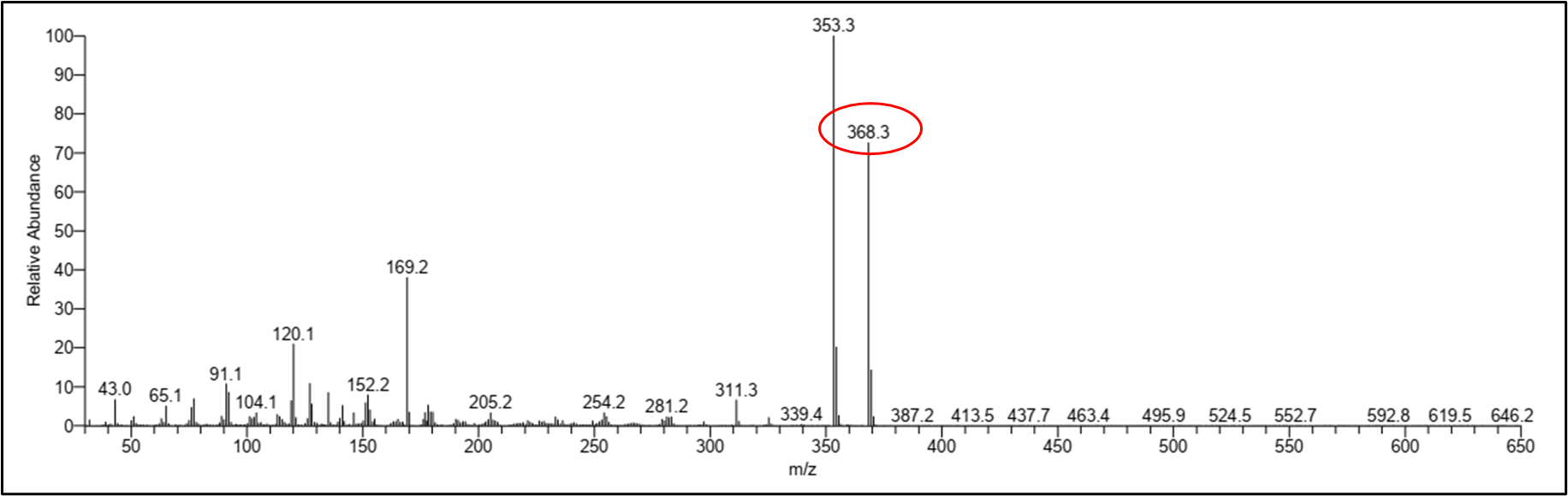


**Figure S3.** Mass spectrum of CA.

Supplement: Supplementary file 1 — Supplementary Figures. [file 41598_2024_61983_MOESM1_ESM.docx]
